# The continuum of ovarian response leading to BIRTH, a real world study of ART in Spain

**DOI:** 10.1186/s40738-020-00081-4

**Published:** 2020-07-29

**Authors:** Marcos Ferrando, Buenaventura Coroleu, Luis Rodríguez-Tabernero, Gorka Barrenetxea, Cristina Guix, Fernando Sánchez, Julian Jenkins, on behalf of the BIRTH study group, Jordi Aragonès Sanahuja, Ramón Aurell Ballesteros, Delia Báez Quintana, Agustín Ballesteros Boluda, Gorka Barrenetxea Ziarrusta, Emilio Bayón Álvarez, Buenaventura Coloreu Lletget, Pilar Conte Martín, José Antonio Domínguez Arroyo, Marcos Ferrando Serrano, Josu Franco Iriarte, José Félix García España, Miguel Ángel García Jiménez, María José Iñarra, Javier Martínez Cortés, Moisés Moreira Pacheco, Cristina Guix Galcerán, Ángel Rocas Huertos, Amelia Rodríguez-Aranda, Luis Rodríguez Tabernero, Bárbara Romero Guadix, Mª. del Carmen Sanabria Rodríguez, Fernando Sánchez Martín, Alejandra Torres Afonso, Margarita Torres Vives, Jesús Zabaleta Jurio

**Affiliations:** 1 IVI-RMA, Bilbao, Spain; 2 Servicio de Medicina de la Reproducción of Hospital Universitari Quirón Dexeus, Barcelona, Spain; 3 Unidad de Reproducción of Hospital Clínico Universitario de Valladolid, Valladolid, Spain; 4 Reproducción Bilbao, Universidad del País Vasco/Euskal Herriko Unibertsitatea, Bilbao, Spain; 5 Barcelona IVF, Barcelona, Spain; 6 Ginemed, Sevilla, Spain; 7 Gedeon Richter/Preglem S.A., Gedeon Richter/ Preglem SA, Route de Frontenex 41A, 1207 Geneva, Switzerland

**Keywords:** IVF, Biosimilar, FSH, Real world study, Combination protocols, Follitropin CRS, Bemfola

## Abstract

**Background:**

The first biosimilar of recombinant follicle stimulating hormone (rFSH) launched in Europe was Bemfola® in 2014 following a clinical development programme demonstrating efficacy and safety to the satisfaction of the European Medicines Agency. Since then the increasing use of biosimilar rFSH has provided the opportunity to study both effectiveness across the whole population and the variation of rFSH use during routine clinical care in a real-world setting in Spain.

**Methods:**

This is a real-world study of 1222 women treated in 26 assisted reproduction treatment centres throughout Spain providing experience of the use of a biosimilar recombinant follicle stimulating hormone in four distinct populations. The four populations studied were poor responders, suboptimal responders, normal responders and oocyte donors. The primary endpoint was the total number of oocytes retrieved. Secondary endpoints included number of days of rFSH stimulation, total dose of rFSH administered, number of MII oocytes, number of fertilized oocytes, quality of embryos, number of embryos transferred, implantation rates, clinical pregnancy rates following embryo transfer, number of multiple pregnancies and number of serious adverse reactions, including moderate-to-severe OHSS.

**Results:**

Differences were seen across the populations both in the characteristics of the women and ART outcomes suggestive of a continuum of fertility prognosis. In the poor responders, suboptimal responders, normal responders and oocyte donor populations the mean age in years was 39.9 (±SD 3.4), 38.4 (±SD 2.9), 34.4 (±SD 3.3) and 26 (±SD 4.6) respectively and number of oocytes retrieved was 4.1 (±SD 2.7), 8.6 (±SD 6.0), 12.2 (±SD 7.2) and 19.5 (±SD 9.5) respectively. The proportion of embryos graded as best quality was 18.5%, 33.0% and 43.8%, and graded as worst quality was 20.4%, 5.8% and 5.8% for poor responders, suboptimal responders and normal responders respectively. In a similar pattern, for poor responders, suboptimal responders and normal responders the implantation rates were 16.0%, (8/50), 22.4% (49/219), 30.6% (97/317) respectively and clinical pregnancy rates were 23.2% (10/43), 30.4% (59/194) and 37.0% (114/308) respectively. Adverse events were reported in only 7 of 1222 women (0.6%).

**Conclusions:**

Overall the results were consistent with the national ART results reported for Spain, hence this study provides reassurance of the clinical effectiveness of a biosimilar rFSH used in a real world setting.

**Trial registration:**

ClinicalTrials.gov identifier - NCT02941341.

## Background

The European IVF-monitoring Consortium (EIM) for the European Society of Human Reproduction and Embryology (ESHRE) estimated that in 2014 one in every 50 children born in Europe were the result of Assisted Reproductive Technology (ART) treatments [1]. Furthermore, in 2014 Spain reported to the EIM a total of 109,275 ART treatments, which was higher than any other European county [1]. Gonadotrophin treatment contributes a significant proportion of the cost of ART, thus the introduction of biosimilars of recombinant follicle stimulating hormone (rFSH) alpha may alleviate the costs of ART improving affordability [2]. The first rFSH biosimilar launched in Europe was Bemfola® in 2014 [3], which has proven popular in Spain [4]. A second rFSH biosimilar, Ovaleap®, was approved in Europe in 2013 and launched in 2016 [3].

The basis of the demonstration of equivalence between a biosimilar and the reference product primarily relies on exhaustive, highly sensitive physicochemical and biological activity comparability assessments later supported by clinical studies leading to a total development time of typically 6 to 12 years [3]. For biosimilar rFSH development the European Medicines Agency (EMA) recommends the “number of oocytes retrieved” as the primary endpoint to demonstrate comparability of clinical efficacy against the reference product, as pregnancy rates are influenced by multiple factors unrelated to ovarian stimulation [5]. The Bemfola® manufacturing conditions, characterization and impurity profile, specifications and stability were all in full compliance with EMA standards [3]. Although biological medicines, both originators and biosimilars, will have inherent variability due to their biological source leading to batch to batch variability, the EMA did not consider there were any differences between Bemfola® and Gonal-f® that would have a significant impact on the product’s safety and efficacy [3]. A pharmacokinetic study of 23 healthy female volunteers revealed no appreciable differences in key pharmacokinetic parameters between Bemfola® and Gonal-f® [6]. The pivotal, randomized, multinational, phase 3 European approval study for Bemfola® versus Gonal-f® was on a population of 372 women aged 20–38 years undergoing upto two IVF/ICSI cycles with typical exclusion criteria including prior excessive or inadequate ovarian response [7]. This study demonstrated equivalence of the two products in the number of retrieved oocytes against a pre-determined clinical equivalence margin of ±2.9 oocytes; Bemfola® yielded 10.8 ± 5.11 oocytes versus Gonal-f® 10.6 ± 6.06 oocytes, mean difference: 0.27, 95% confidence interval: − 1.34, 1.32. Also a similar clinical pregnancy rate per embryo transfer was observed in first and second cycles (Bemfola®: 40.2 and 38.5% vs Gonal-f®: 48.2 and 27.8%, respectively). No difference was seen in severe ovarian hyperstimulation syndrome rates between treatment groups (Bemfola: 0.8%; Gonal-f: 0.8%) nor overall safety profiles with no evidence of immunogenicity in either group.

Beyond the demonstration of efficacy in carefully controlled phase 3 clinical trials on selected populations, the value of assessing the effectiveness of new treatments across the whole population undergoing routine clinical care in a real-world setting is becoming increasingly recognised [8]. The EU Medicines Agencies Network Strategy to 2020 drew attention to the value of real-world studies to assess effectiveness of new drugs in a real world setting and even in sub populations [9]. The BIRTH study was a post approval, non-interventional study on the use of Bemfola®, assessing the effectiveness of Bemfola® in 4 different populations undergoing ART in Spain. Furthermore, the BIRTH study provides a snap shot of ART practice in Spain revealing important differences in the populations undergoing ART.

## Methods

### Study design

The BIRTH study was a post EMA approval, non-interventional study of women who had undergone oocyte retrieval after ovarian stimulation with Bemfola® with GnRH antagonist pituitary-suppression, either as part of an autologous in vitro fertilisation (IVF) / intracytoplasmic sperm injection (ICSI) treatment cycle or for oocyte donation. Other than the latter requirements treatment was according to the centres’ standard of care. In 28% of autologous embryo transfers all embryos had been cryopreserved and embryo transfer delayed. The study was carried out in 26 Spanish Assisted Reproduction centres, both public and private, belonging to 10 regions throughout Spain.

The study was designed by the sponsor (Finox Biotech Iberia, S.L., which in June 2016 was acquired by Gedeon Richter Ibérica S.A) supported by a group of Spanish ART experts. The study was approved by the Ethics Committee of Clinical Research of Euskadi (CEIC-E). Enrolment was conducted from September 2016 until April 2018 with the data collected and analysed by Dynamic, Azcona, 3,128,028 Madrid, Spain. All authors were involved in the preparation of the paper and validate the accuracy of the data.

### Study population

Women aged ≥18 years at recruitment, either patients undergoing an IVF/ICSI procedure or egg donors who completed a controlled ovarian stimulation receiving at least 5 doses of Bemfola® were included in the study. Pituitary suppression was achieved by administering GnRH antagonists. All participating women signed an Informed Consent Form, prior to any data collection. Women reporting hypersensitivity to follitropin alfa or to any of the excipients of Bemfola® were excluded. The presence of a pituitary or hypothalamic tumour was also an exclusion criteria.

Patients were divided into four populations (poor responders, suboptimal responders, normal responders and oocyte donors) defined as follows. Poor responders were defined according to the Bologna definition of having at least two of the following criteria: advanced reproductive age (≥40 years) or any other risk for poor ovarian response; previous poor ovarian response (≤3 oocytes with a conventional protocol of stimulation, and abnormal ovarian reserve results - antral follicle count < 5 to 7 follicles, anti-mullerian hormone (AMH) < 0.5–1.1 ng/ml [10]. Suboptimal responders were defined according to two subsets; firstly, women aged < 38 years with either a previous poor ovarian response (≤3 oocytes) with a conventional protocol or abnormal ovarian reserve tests (AFC < 5 follicles, AMH < 0,5 ng/ml). The second subset of patients defined as suboptimal responders were those women aged > 37 years without any of the features described above, but according to routine clinical practice were stimulated with > 225 IU of FSH in combination with LH activity from human menopausal gonadotropin (HMG) or recombinant luteinising hormone (rLH). Normal responders were defined as women aged < 38 years with no risk factors of poor ovarian reserve (AMH < 1.5 ng/ml, endometriosis grade I-II, previous poor ovarian response) and women with high ovarian reserve, including women with polycystic ovary syndrome.

### Measurements

The primary endpoint was the total number of oocytes retrieved. Secondary endpoints included number of days of rFSH stimulation, total dose of rFSH administered, number of MII oocytes, number of fertilized oocytes, quality of embryos, number of embryos transferred, implantation rates, clinical pregnancy rates following embryo transfer and number of multiple pregnancies. To support routine post marketing pharmacovigilance monitoring moderate-severe ovarian hyperstimulation syndrome (OHSS) and serious adverse reactions were recorded.

### Statistical analysis

Since the purpose of this study was purely descriptive, no formal sample size calculations based on comparative hypothesis testing were conducted but rather the sample size was based on ensuring a suitable size to describe the effectiveness of Bemfola® in routine use. As this was a real world study doctors were free to treat patients as they felt appropriate and there was no randomisation nor stratification into study groups. It would thus be anticipated that there would be considerable heterogeneity within groups, which may be subject to a number of biases. Accordingly, the data is only provided descriptively using appropriate methodology to avoid misleading the reader with any attempt at comparative analyses, which would be inherently flawed.

## Results

A total of 1222 women were available for data analysis divided into four populations: poor responders (*n* = 96), suboptimal responders (*n* = 301), normal responders (*n* = 386) and oocyte donors (*n* = 439). Data were not collected from the oocyte recipient treatment cycles and patient flow for each population throughout treatment is summarised in Table 1, which also provides details of the completeness of the data.

**Table 1:** Patient Flow and completeness of data recording

Populations	Total	Poor Responders	Suboptimal Responders	Normal Responders	Oocyte Donors
All evaluable patients (n)	1222	96	301	386	439
Cycles proceeding to oocyte retrieval (n)	1169	88	274	378	429
Number of cycles where oocyte number recorded (n)	1169	88	274	378	429
Cycles with Oocytes fertilized (n)	642	58	240	344	NA
Cycles with Embryos Transferred (n)	546	43	194	309	NA
Number of cycles where number of embryos transferred recorded (n)	362	33	135	194	NA
Number of embryo transfer cycles where outcome of cycle known (n)	533	41	191	301	NA
Cycles resulting in clinical Pregnancy (n)	183	10	59^a^	114	NA
Number of cycles where number of gestation sacs recorded (n)	182	10	59	113	NA

^a^*In addition, there was one ectopic pregnancy*

*NA = not appropriate*

**Fig. 1:**
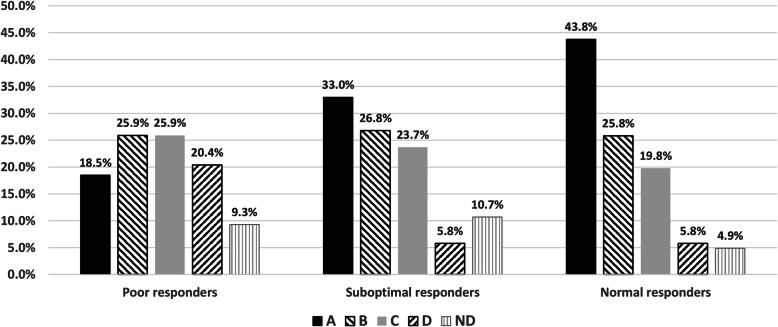
Embryos graded according to the “Criterios ASEBIR de Valoración Morfológica de Oocitos, Embriones Tempranos y Blastocistos Humanos”; A = top quality; B = good quality (not for elective single embryo transfer); C = impaired embryo quality; D = do not recommend to transfer (includes all multinucleated embryos); ND = not classified [10, 11]

As expected, there were differences in demographic profile among the assessed populations of women (Table 2). Despite higher total amount of gonadotrophin stimulation in the poor responder and suboptimal responders, these populations produced both fewer total number of oocytes and fewer metaphase two oocytes compared to the normal responders and oocyte donors (Table 3). Despite differences in the number of fertilised oocytes between the populations the eventual number of embryos transferred did not differ greatly between them (Table 4). However, the proportion of embryos graded according to the criteria of the Asociación para el Estudio de la Biología de la Reproducción (ASEBIR) [11, 12] as best quality was 18.5%, 33.0% and 43.8% and worst quality was 20.4%, 5.8% and 5.8% for poor responders, suboptimal responders and normal responders respectively (Fig. 1). Further, the normal responder population had both a better implantation rate and more pregnancies compared to the poor responder and suboptimal responder populations (Table 5).

**Table 2:** Demographic and baseline details of all patients

	All women (*n* = 1222)	Poor Responders (*n* = 96)	Suboptimal responders (*n* = 301)	Normal Responders (*n* = 386)	Oocyte Donors (*n* = 439)
Age (years): mean (SD)	32.8 (6.5)	39.9 (3.4)	38.4 (2.9)	34.4 (3.3)	26 (4.6)
Weight (Kg) mean (SD)	62.3 (9.9)	64.1 (9.8)	63.8 (9.7)	63.2 (9.7)	60.2 (9.6)
Cause of infertility (% vs total patients)					
Ovulatory		14.0%	9.7%	11.3%	
Tubal		9.7%	9.0%	11.6%	
Male		10.8%	19.7%	37.3%	
Idiopathic		3.2%	14.0%	19.8%	
Mixed / Other		62.3%	47.6%	20.0%	
Baseline FSH IU/L (SD)	7.4 (2.9)	8.9 (4.2)	8.0 (3.7)	6.9 (2.0)	5.4 (2.8)
AMH mean ng/ml (SD)	2.8 (2.6)	0.9 (0.7)	1.9 (1.8)	3.4 (2.8)	4.6 (2.5)

Note: Means & percentages are based on the number of patients with non-missing data

**Table 3:** Results of ovarian stimulation for patients reaching oocyte retrieval

	All women (*n* = 1169)	Poor Responders (*n* = 88)	Suboptimal responders (*n* = 274)	Normal Responders (*n* = 378)	Oocyte Donors (*n* = 429)
Ovarian stimulation duration days mean (±SD)	9.4 (2.2)	10.1 (2.6)	9.6 (2.4)	9.4 (2.2)	9.3 (1.9)
Total dose of r-FSH administered IU mean (± SD)	2093.4 (698.3)	2423.2 (937.1)	2083.4 (749.6)	2040.3 (752.9)	2075.7 (509.4)
No. of retrieved oocytes mean (± SD)	13.4 (9.4)	4.1 (2.7)	8.6 (6.0)	12.2 (7.2)	19.5 (9.5)
No. of MII oocytes mean (± SD)	10.7 (7.5)	3.5 (2.3)	6.5 (4.7)	9.8 (6.2)	15.4 (7.9)

Note: Means & percentages are based on the number of patients with non-missing data

**Table 4:** Results of Insemination

	All women (*n* = 642)	Poor Responders (*n* = 58)	Suboptimal responders (*n* = 240)	Normal Responders (*n* = 344)
Number of fertilized oocytes mean (± SD)		2.3 (1.8)	4.5 (3.6)	6.8 (4.8)
Fertilization rate (%) (number of fertilized oocytes / number of inseminated or microinjected MII oocytes)		71.7%	68.4%	72.4%
Number of embryos transferred				
1 n (%)	120 (33.1%)	12 (36.4%)	49 (36.3%)	59 (30.3%)
2 n (%)	239 (65.8%)	21 (63.6%)	83 (61.5%)	135 (69.2%)
3 n (%)	3 (0.85)	0 (0%)	3 (2.2%)	0 (0%)

Note: Means & percentages are based on the number of patients with non-missing data

**Table 5:** Pregnancy outcome

	All women	Poor Responders	Suboptimal responders	Normal Responders
Implantation rates (Total number of sacs / Total number of embryos transferred)	26.3% (154/586)	16.0% (8/50)	22.4% (49/219)	30.6% (97/317)
Clinical pregnancy (Total clinical pregnancies / Total number of women with embryos transferred)	33.6% (183/545)	23.2% (10/43)	30.4% (59/194)	37.0% (114/308)
Number of gestation sacs (% vs total sacs)				
1	156 (85.7%)	10 (100%)	53 89.8%)	93 (82.3%)
2	26 (14.3%)	0 (0%)	6 (10.2%)	20 (17.7%)

Note: Means & percentages are based on the number of patients with non-missing data

The data was further analysed with respect to the use of rFSH alone in monotherapy protocols or with a product having LH activity in combination protocols (Tables 6, 7 and 8). Across the full study population, combination protocols were used in 28.7% of women less than 35 years and 59.6% of women 35 years of age or older. Within each of the populations, combination protocols were used in 54.5% of poor responders, 67.5% of suboptimal responders, 38.1% of normal responders and 25.9% of oocyte donors. Within each population the number of retrieved oocytes, implantation rates and clinical pregnancy rates tended to be lower with combination protocols than monotherapy protocols (Tables 7 and 8).

**Table 6:** Age groups Monotherapy vs Combination Protocol

Age		Monotherapy *n* = 702 (57.4%)	Combination^**a**^ *n* = 520 (42.6%)	Total
< 35 years	n (%)	481 (71.3%)	194 (28.7%)	675
35- < 38 years	n (%)	114 (47.5%)	126 (52.5%)	240
38- < 40 years	n (%)	49 (30.4%)	112 (69.6%)	161
> = 40 years	n (%)	58 (39.7%)	88 (60.3%)	146

^**a**^ addition of recombinant LH or HMG to rFSH

**Table 7:** Results of ovarian stimulation – Monotherapy vs Combination Protocol

	All women	Poor Responders	Suboptimal responders	Normal Responders	Oocyte Donors
**Monotherapy**	(*n* = 681)	(*n* = 40)	(*n* = 89)	(*n* = 234)	(*n* = 318)
No. of retrieved oocytes mean (± SD)	15.1 (9.4)	3.7 (2.6)	9.9 (7.2)	13.6 (7.6)	19.1 (9.5)
**Combination**	(*n* = 488)	(n = 48)	(*n* = 185)	(*n* = 144)	(*n* = 111)
No. of retrieved oocytes mean (± SD)	11.2 (8.5)	4.6 (2.7)	7.9 (5.2)	10.1 (6.1)	20.8 (9.4)

Note: Means & percentages are based on the number of patients with non-missing data

**Table 8:** Pregnancy outcome – Monotherapy vs Combination Protocol

	All women	Poor Responders	Suboptimal responders	Normal Responders
**Monotherapy**				
Implantation rates	32.8%	25.0%	22.9%	35.7%
(Total number of sacs / Total number of embryos transferred)	(79/241)	(2/8)	(11/48)	(66/185)
Clinical pregnancy rate	38.8%	27.3%	35.6%	40.4%
(Total clinical pregnancies / Total number of women with embryos transferred d)	(100/258)	(3/11)	(21/59)	(76/188)
**Combination**				
Implantation rates	21.7%	14.3%	22.2%	23.5%
(Total number of sacs / Total number of embryos transferred)	(75/345)	(6/42)	(38/171)	(31/132)
Clinical pregnancy rate	28.9%	21.9%	28.1%	31.7%
(Total clinical pregnancies / Total number of women with embryos transferred)	(83/287))	(7/32)	(38/135)	(38/120)

Note: Means & percentages are based on the number of patients with non-missing data

### Pharmacovigilance data

There were only 7 out of the 1222 women (0.6%), in whom adverse events were reported. Ovarian hyperstimulation syndrome (OHSS) of at least moderate severity occurred in 1 case in the suboptimal responder population and in 4 cases of the normal responder population, of which 1 case was reported as severe OHSS. In addition, two serious adverse events were reported. One patient (out of 386), in the normal responder population, reported severe pain after egg retrieval. One oocyte donor (out of 439 women) reported a Bell’s palsy, which was considered unrelated to medication. All adverse events reported during the study were collated with adverse events reported directly to the Gedeon Richter pharmacovigilance department and the consolidated pharmacovigilance data is presented in this manuscript.

## Discussion

By studying four populations of women (poor responders, suboptimal responders, normal responders and oocyte donors) the BIRTH study shows a clear and related continuum of ovarian response and fertility among women. The majority of patients were not classified consistently across all centres with a clear sole cause of infertility illustrating the complexity of understanding the cause of infertility and agreeing to a common diagnostic terminology [13]. Nevertheless, there were obvious differences seen between the populations of women in this study. The populations showed both quantitative and qualitative differences in terms of oocyte number achieved and embryo quality, respectively, associated with striking differences in implantation rates and pregnancy rates. This illustrates the value of assessing a new drug across a wide range of patients treated in diverse ways in real-world situations, as randomised control trials will include patients of often very limited prognoses, which may not be adequately balanced between study groups following randomisation, particularly if the sample size is relatively low, and which may not reflect the potential populations in real clinical practice. Furthermore, the study confirmed that Bemfola® was able to meet the treatment requirements for all patient types whether using monotherapy or combination protocols.

Although this study adopted the Bologna criteria [10] to define the poor responder population, the limitations of this definition are acknowledged, which is why a further population of suboptimal responders was included in this study. Although the concept of poor ovarian response has been recognised for a very long time, in 2011 an enormous variability of the definitions of poor ovarian response in the literature was reported [14]. Thus in 2011 the European Society of Human Reproduction and Embryology (ESHRE) established strict criteria (Bologna consensus) to define poor ovarian response thereby establishing a more homogenous population to support research [10]. Although the Bologna criteria was a crucial step towards defining poor ovarian response, it became clear that even when using the Bologna criteria, the poor ovarian response population remained heterogeneous primarily because the criteria did not adequately take the age-related impact on oocyte quality into consideration, which significantly impacts success rates [15]. In 2016 a group of reproductive endocrinologists and scientists gathered to further refine the definition of poor ovarian response [16]. As a result, the new POSEIDON (Patient-Oriented Strategies Encompassing Individualized Oocyte Number) classification was developed, providing a more detailed classification to reduce the heterogeneity of the Bologna criteria [17]. When considering the definition of “poor ovarian response”, the introduction of the follicular output rate (FORT) [18] provides an alternative measure of ovarian response to exogenous stimulation by assessing the ratio between the number of pre-ovulatory follicles obtained in response to gonadotropin administration and the pre-existing pool of small antral follicles. The FORT concept might even be taken a step further, including also the ratio between the final number of oocytes retrieved correlated to the antral follicle count (AFC) to measure successful ovarian response. As an example, a patient with a poor ovarian reserve who finally ends up with 70% of the antral follicles, resulting in retrieved oocytes has a high FORT, and in reality, a good ovarian response to stimulation regardless of the total number of oocytes retrieved. Evidentially, although the concept is clear, the precise definition of poor ovarian response remains a challenge. This paper presents two populations of relatively less than normal ovarian response demonstrating relatively poorer outcomes supportive of a heterogenous, continuum of ovarian responsiveness with associated differing prognoses rather than a discrete group of poor ovarian response patients.

No lack of efficacy nor pharmacovigilance signals were revealed in this real-world study of the use of this new product and in fact the differences between patient groups was far more relevant to whether a patient was successful with ART or suffered an adverse event than the choice of gonadotrophin. Furthermore, the pregnancy rates in the BIRTH study were consistent to the overall pregnancy rates per embryo transfer reported in 2017 to the national Spanish ART registry of 42%, 36.5% and 25.6% for IVF (*n* = 6473) and 44.5%, 36.3% and 21.2% for ICSI (*n* = 43,790) according to ages < 35 years, 35–39 years and ≥ 40 years respectively [19]. The national overall multiple embryo transfer rates for Spain were 49% in 2016 and 44% in 2017 [19].

One area of particular interest explored in this study was the use of FSH with a product providing LH activity in what is referred to as combination, combo or mixed protocols. The study showed combination protocols are commonly used in Spain, particularly in patients over 35 years of age or among patients anticipated to have a reduced response to ovarian stimulation. Although this study is not a randomised control study and there is considerable heterogeneity in terms of the details of the combination protocols used at different centres, the results are interesting to consider in the context of the literature and recent guidance on ovarian stimulation for IVF/ICSI from the European Society of Human Reproduction and Embryology [20].

In each BIRTH study population the implantation and pregnancy rates were numerically lower with combination protocols than with mono therapy. Although statistical comparisons of these results would be inappropriate as patients allocated to combination protocols might be of a poorer prognosis even within patient populations, at the very least the results do not suggest any benefit for combination protocols in any of the patient populations studied. The ESHRE ovarian stimulation for IVF/ICSI guidelines similarly could not identify any groups that would benefit from the addition of LH activity to FSH stimulation, other than the rare cases of WHO-I anovulatory patients. The ESHRE ovarian stimulation for IVF/ICSI guidelines specifically commented there was no evidence of a beneficial effect on live birth rates for the LH supplementation of rFSH in poor responders or women of advanced age. In addition and of note, the ESPART Study in “poor responders” suggested fewer oocytes with LH and no benefit in pregnancy rates [21] and a meta-analysis of 5840 patients in twenty-nine studies confirmed LH reduced number of oocytes when added to FSH [22]. A further large randomised trial, MEGASET, showed that not only was the addition of LH activity associated with fewer oocytes, but there was no benefit in the quality of oocytes, embryos and blastocysts [23].

This evidence of clinical effectiveness from this study is particularly important as further to Bemfola® being approved by the European Medicines Agency in 2014 as a biosimilar of follitropin alpha, in 2017 the Bemfola® drug substance batch number T128/FSH/B1 was adopted as the European Follitropin Chemical Reference Substance (CRS) [24, 25]. The drug substance is essentially the active pharmaceutical ingredient (API) or the ‘naked’ drug without excipients. The API is what will have a therapeutic effect inside the body as opposed to the excipients that serve to package and deliver the API. The drug product is the formulated mixture of the drug substance and excipients which results in the final marketed product. The quality and consistency of any rFSH drug substance prior to release of the final rFSH drug product may be assessed against the Follitropin CRS according to the European Pharmacopoeia (Ph.Eur) of the European Directorate for the Quality of Medicines and Healthcare (EDQM).

Although a real world study has the advantage of reporting on the whole highly heterogeneous population receiving a very diverse range of clinical ART practices, inevitably this leads to study limitations of low internal validity, lack of quality control and susceptibility to multiple sources of bias. Further the BIRTH study did not present spontaneous abortion rates, live birth rates and rates of congenital anomalies. Hence, real world studies such as the BIRTH study should be viewed as part of a wider body of evidence to guide clinical practice.

## Conclusions

Fertility is a continuum from poor responders, suboptimal responders, normal responders through to fertile oocyte donors. Bemfola® produced the anticipated number of oocytes according to patients’ populations with adequate embryo quality and pregnancy rates in a real world setting which were consistent with Spanish national ART reports. In addition a low rate of adverse reactions were reported. The results seen regarding the use of combination protocols reflect the medical literature and recent guidance from ESHRE questioning the value of adding LH activity to gonadotrophin stimulation protocols [20].

## Data Availability

The datasets used in the current study are available from the corresponding author on reasonable request.

## Abbreviations

AFC: Antral follicle count
AMH: Anti-Müllerian hormone
ART: Assisted Reproductive Technology
ASEBIR: Asociación para el Estudio de la Biología de la Reproducción
CRS: Chemical Reference Substance
EDQM: European Directorate for the Quality of Medicines and Healthcare
EMA: European Medicines Agency
ESHRE: European Society for Human Reproduction and Embryology
FSH: Follicle stimulating hormone
ICSI: Intracytoplasmic sperm injection
IVF: In vitro fertilization
LH: Luteinizing hormone
OHSS: ovarian hyperstimulation syndrome
Ph.Eur: European Pharmacopoeia
SD: Standard deviation
SEF: Sociedad Española de Fertilidad
